# A novel Tn*1696*-like composite transposon (Tn6404) harboring *bla*_IMP-4_ in a *Klebsiella pneumoniae* isolate carrying a rare ESBL gene *bla*_SFO-1_

**DOI:** 10.1038/s41598-017-17641-2

**Published:** 2017-12-11

**Authors:** Kai Zhou, Wei Yu, Ping Shen, Haifeng Lu, Baohong Wang, John W. A. Rossen, Yonghong Xiao

**Affiliations:** 1 State Key Laboratory for Diagnosis and Treatment of Infectious Diseases, Collaborative Innovation Center for Diagnosis and Treatment of Infectious Diseases, the First Affiliated Hospital of Medicine School, Zhejiang University, Hangzhou, China; 2 Department of Medical Microbiology, University of Groningen, University Medical Center Groningen, Groningen, Netherlands; 3 Department of Infectious Diseases, Zhejiang Provincial People’s Hospital, Hangzhou, China

## Abstract

Genetic determinants of a clinical *Klebsiella pneumoniae* isolate (KP1814) coproducing IMP-4 and a rare ESBL gene SFO-1 was investigated. KP1814 belongs to a novel sequence type (ST) assigned to ST2270. WGS identified four circular DNA sequences in KP1814, including two multidrug-resistance (MDR) plasmids, one virulence plasmid, and one circular form. The MDR plasmid pKP1814-1 (299.9 Kb) is untypeable, and carries two large mosaic multiresistance regions (MRRs). *bla*_SFO-1_ and *bla*_IMP-4_ co-exists on MRR1, and *bla*_SFO-1_ is associated with an IS/Tn-independent genetic context. *bla*_IMP-4_ is carried by a novel In804-like integron (*intlI*-*bla*_IMP-4_-*Kl.pn.I3*-*qacG2*-*aacA4*-*catB3*∆) associated with a novel Tn*1696*-like transposon (designed Tn*6404*) flanked by IS*5075*. The other MDR plasmid pKP1814-3 is a 95,701-bp IncFII plasmid, and is a hybrid of a *Shigella flexneri* plasmid pSF07201 and an *E*. *coli* plasmid pCA08. All resistance genes of pKP1814-3 were detected in a ~16-kb IS*26*-flanked composite transposon carried by a Tn*5396* transposon. The circular form (18.3 Kb) was composed of two parts belonging to pKP1814-1 and pKP1814-3, respectively. The plasmid pKP1814-2, carrying multiple virulence factors, encodes IncFIB_K_ and IncFII_K_ replicons with a size of 187,349 bp. The coexistence of MDR and virulence plasmids largely enhances the bacterial fitness in the host and environment.

## Introduction

SFO-1 is a rare class A ESBL first identified on a self-transferable plasmid in a clinical *Enterobacter cloacae* isolate from Japan in 1999^1^. By contrast to the known plasmid-borne β-lactamases which are produced constitutively, SFO-1 is induced by imipenem, and can hydrolyze most β-lactams except cephamycins and carbapenems^2^. The *bla*_SFO-1_ gene was later detected in an outbreak clone of multidrug-resistance (MDR) *E*. *cloacae* in Spain^3^, and in three *Klebsiella pneumoniae* and one *Escherichia coli* clinical isolates in China^4,5^. However, the complete structure of *bla*_SFO-1_-harboring plasmids is still unknown.

IMP-4 was first detected in Hong Kong in 2001^6^, and later widely disseminated in Australia and the mainland of China^7^. The spread of IMP-4 is frequently associated with class 1 integrons carried by plasmids^8,9^. In China, plasmid-borne *bla*_IMP-4_ has been sporadically reported in different provinces/cities (Chongqing, Hubei, Fujian, Zhejiang, Guangdong, Shanghai and Tianjin^10–16^. However, few studies have reported the full structure of *bla*_IMP-4_-carrying plasmids detected in China^16–18^, largely limits our understanding on the dissemination mechanism of *bla*_IMP-4_. In this study, we report a carbapenem-resistant *K*. *pneumoniae* isolate that simultaneously carries *bla*_IMP-4_ and *bla*_SFO-1_ genes. The plasmid content and genetic determinants of the isolate were comprehensively analyzed to gain a better understanding of the spread mechanism of *bla*_IMP-4_ and *bla*_SFO-1_ genes in China.

## Materials and Methods

### Strain collection

The community-associated *K*. *pneumoniae* strain KP1814 was collected in the frame of a national-wide survey for antibacterial resistance among outpatients with community-associated infections^19^. This strain was isolated from an outpatient (the type of clinical specimen was unknown) in a secondary hospital in mid-south of China (Hubei province) in 2011.

### Antimicrobial Susceptibility Testing

Antimicrobial susceptibility was determined using Vitek-2 with card GN-13 (Biomerieux, Marcyl’Etoile, France) and was interpreted according to the clinical breakpoints defined by EUCAST-v7.0 (http://www.eucast.org/clinical_breakpoints/). ESBL phenotype was confirmed by the double-disc synergy test (DDST) using cefotaxime (30 μg) and ceftazidime (30 μg) with clavulanate (10 μg) discs.

### Plasmid analysis

Azide-resistant *E*. *coli* J53 was used as the recipient for the conjugative transfer of plasmids. Transconjugants were selected on MH media with sodium azide (100 µg/µl) and gentamicin (10 µg/µl) or ceftazidime (8 µg/µl). The selected transconjugants were later confirmed by PCR and susceptibility testing. The plasmid size was estimated by S1 nuclease pulsed-field gel electrophoresis (PFGE).

### Whole-genome sequencing (WGS) and data analysis

Genomic DNA was extracted using Gentra Puregene Yeast/Bacteria Kit (Qiagen, Hilden, Germany). The genome was sequenced by Illumina Hiseq2500 (Illumina, San Diego, CA, USA) using a 2 × 125-bp pair-end libraries, and was further scaffolded by Pacbio RS II platform (Pacific Biosciences, California, US) using a 10-Kb library. *De novo* assembly for reads yielded by Hiseq2500 was done by CLC Genomics Workbench v8.0 (QIAGEN, Hilden, Germany) after quality trimming (Qs ≥ 20), and the scaffolding was performed by SSPACE standard version 3.0 with default settings^20^. Further gaps within scaffolds were closed using GapFiller with default settings^21^. Annotation was performed using the RAST server (rast.nmpdr.org) followed by manual curation using BlastP and ISfinder (https://www-is.biotoul.fr); multilocus sequence typing (MLST), resistome analysis and plasmid typing was done by uploading sequences to the CGE server (https://cge.cbs.dtu.dk).

### Nucleotide Sequence Genbank Accession Numbers

The three plasmids and the circular form have been deposited at DDBJ/EMBL/GenBank under the accession of KX839207- KX839210.

## Results and Discussion

### The characterization of KP1814

KP1814 was resistant to numerous drugs, including ertapenem, and remained susceptible to amikacin, ciprofloxacin, and levofloxacin (Table S1). KP1814 showed an ESBL-negative phenotype by double-disc synergy test. However, WGS detected a rare ESBL gene *bla*_SFO-1_ which was overlooked using routine PCRs^19^. A carbapenemase gene *bla*_IMP-4_ and 23 acquired drug-resistant genes were further detected, including *aac(3′)-IId* (2 copies), *aacA4*, *strA*, *strB*, *aac(6*′*)-Ib-*cr (2 copies), and *aadA5* (2 copies) for aminoglycoside resistance; *bla*_TEM-1b_ and *bla*_OXA-1_ for β-lactam resistance; *mph(A)* (3 copies) for macrolide; *catB3* and *catB3*∆ for phenicol; *arr-3* for rifampicin; *sul1* (3 copies) for sulphonamide; *dfrA17* (2 copies) for trimethoprim. This genotype can fully explain the resistance profile observed. KP1814 additionally carries *bla*_SHV-11_ and *fosA* on the chromosome. KP1814 was assigned to the novel sequence type (ST) 2270 (2-3-1-20-61-4-133). S1 PFGE showed three clear bands with sizes approximately 300 kb, 180 kb, and 90 kb (data not shown).

The genome of KP1814 was completed to dissect its plasmid content. This resulted in one complete chromosome, 3 complete circular plasmids (pKP1814-1, pKP1814-2, and pKP1814-3) and one circular form with size of 299.9Kb, 187.3Kb, 95.6Kb, and 18.2Kb, respectively. This is in concordance with the PFGE results except the circular form that was not detected on the gel.

### The characterization of pKP1814-1

The *bla*_IMP-4_ and *bla*_SFO-1_ genes co-locate on pKP1814-1. To the best of our knowledge, this is the first report on the co-existence of *bla*_SFO-1_ and *bla*_IMP-4_. pKP1814-1 was able to be transferred to the azide-resistant *E*. *coli* J53 via conjugation, and the conjugation frequency was estimated of ~5 × 10^−5^ per donor cell.

**Figure 1:**
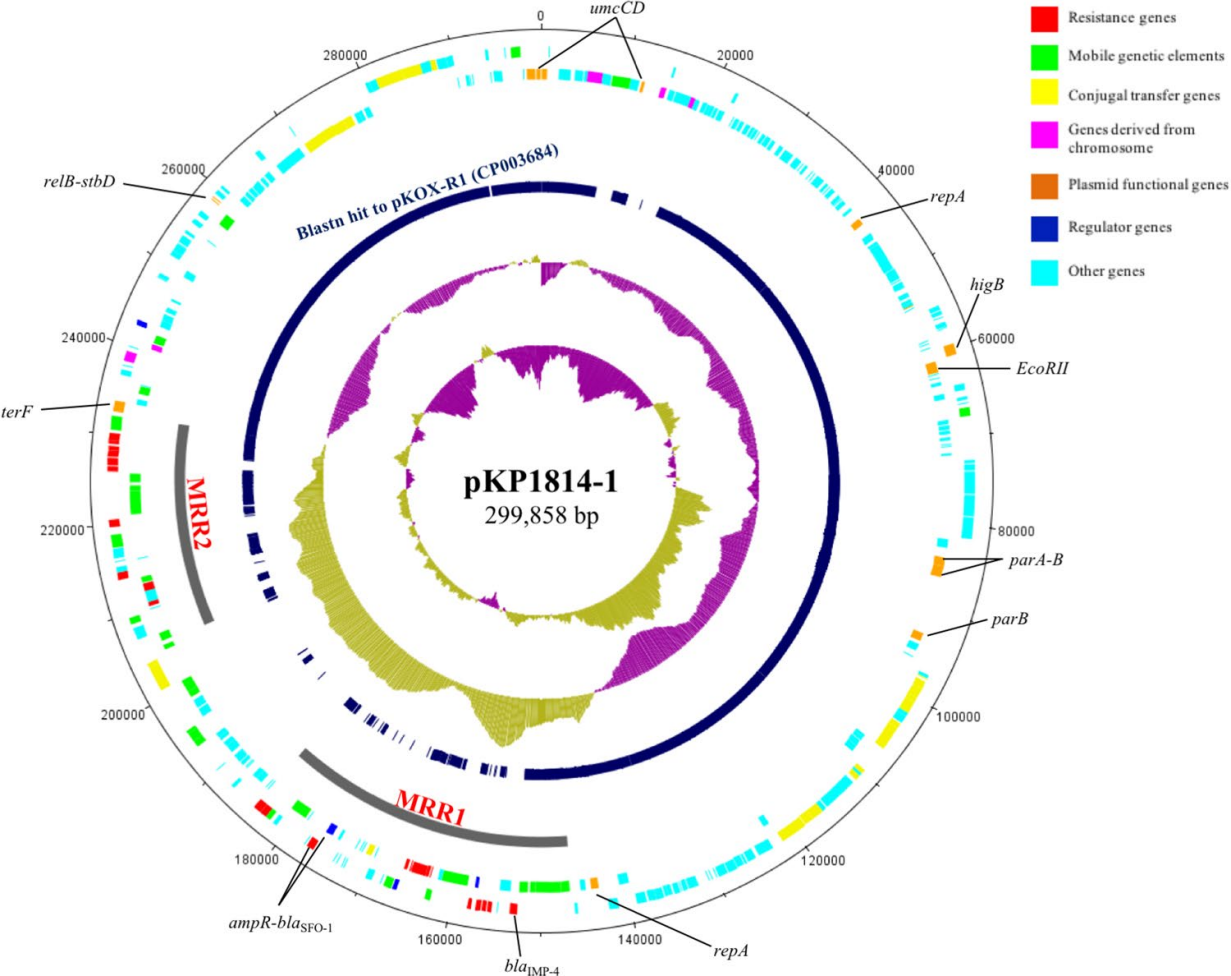
The genetic map of pKP1814-1. A circular representation of pKP1814-1 was generated by using DNA plotter. From the inside outward, the first, second, and third circle show GC skew, GC content, and the regions with over 50% nucleotide identity to pKOX-R1(CP003684), respectively. The fourth and fifth circle show the genetic features in counterclockwise and clockwise directions, respectively. Genes are classified by different colours as shown in the legend, and the gene names of plasmid backbone are shown. Two MRRs are marked by gray arches.

pKP1814-1 is a 299,858-bp plasmid (Fig. 1), and is untypeable by using PlasmidFinder. This is different from previously reported *bla*_IMP-4_-encoding plasmids that are frequently associated with incompatibility groups IncL/M, IncA/C and IncHI2 identified in Australia^22^, and IncN in China^16–18^. pKP1814-1 shares a high homology with an IncA/C-IncH plasmid (the replicon type is IncU reanalyzed by PlasmidFinder in this study) pKOX-R1 (CP003684; 77% coverage, >98% nucleotide identity)^23^, and an IncH plasmid pRpNDM1-1 (JX515588; 66% coverage, 99% nucleotide identity). The shared backbone between pKP1814-1 and pKOX-R1 is highly conserved with 82-bp difference (>99.9% nucleotide identity), including the replicon gene *repA*, *umuC/D* (for DNA repair), *higB* (encoding toxin), *parA/B* (encoding partitioning proteins), *relB*/*stbD* (for plasmid stability), conjugative transfer gene clusters dispersed in different locations (*traIDJLEKBVCWUNX*, *traCBP*, and *traFHG*) and other genes encoding function/hypothetical proteins (Fig. 1). Notably, pKOX-R1 and pRpNDM1-1 were identified in Taiwan and mainland China, respectively, suggesting a circulation of such plasmids in that geographic region. However, various replicon types carried by these plasmids highly challenges the epidemiological investigations.

### The multi-resistance regions (MRRs) of pKP1814-1

**Figure 2:**
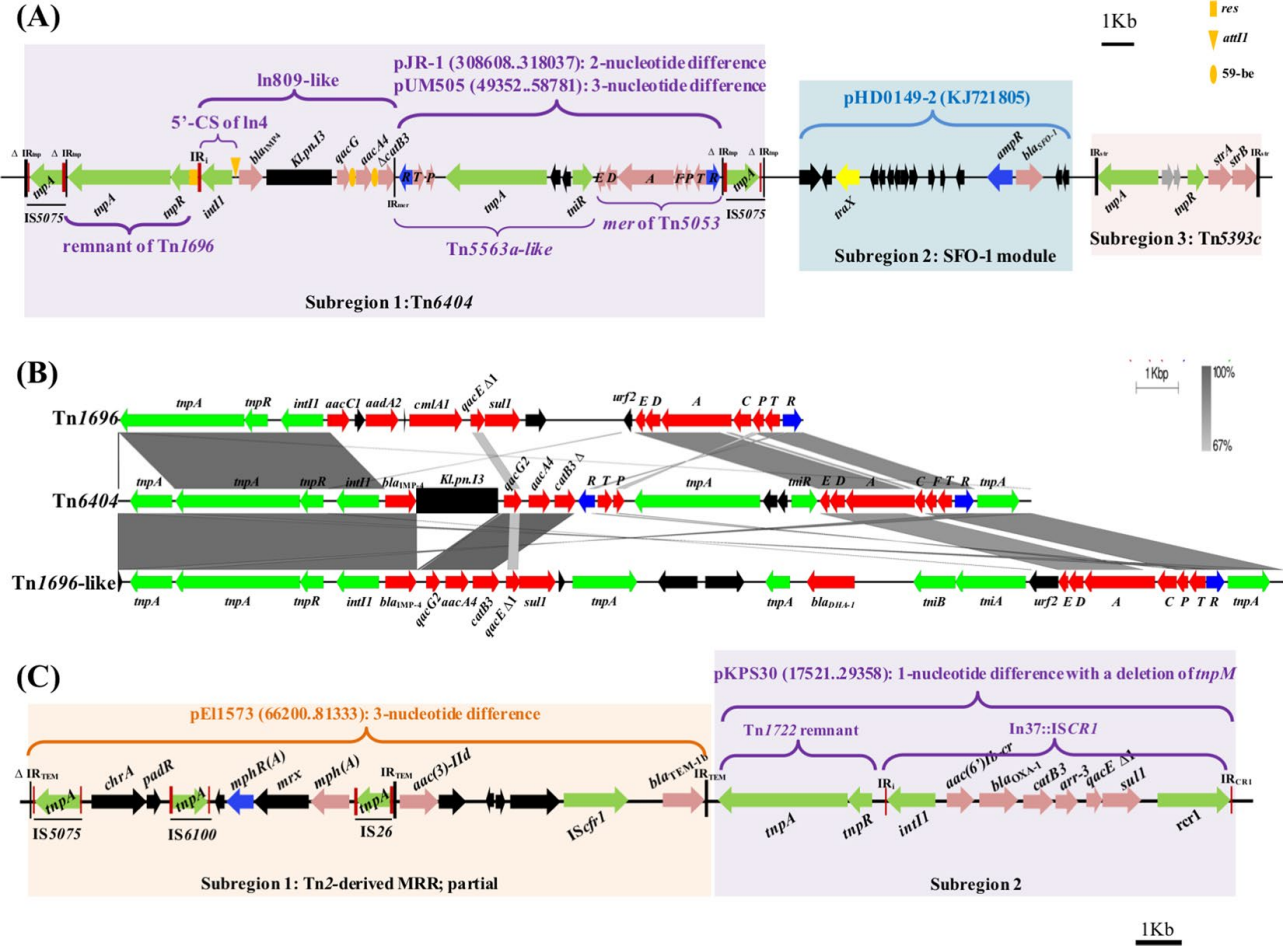
Features of the two MRRs of pKP1814-1. (**A**) The structure of MRR1. Three subregions of MRR1 are highlighted in different colours. Black tall bars represent the IRs of Tns. The 38-bp IR (IR_tnp_) of Tn*1696* is interrupted into two parts by the insertion of IS*5075*. Shorter red bars represent the IRs of IS/In. The group II intron *Kl.pn.I3* is shown as a black rectangle. The genes of mer operon are labeled with their letter, and the other genes are marked in black; (**B**) Comparison of Tn*6404*, Tn*1696* and Tn*1696*-like. The Tn*1696*-like transposon is detected on pIMP-PH114 (96158..123870); (**C**) The structure of MRR2. Two subregions of MRR2 are highlighted in different colours. The IR name of the subregion 1 is derived from pEl1573 (JX101693).

All drug-resistant genes of pKP1814-1 were detected on two MRRs (Fig. 1). MRR1 is bracketed by an interrupted IR_tnp_ of Tn*21* (22 bp) and an 81-bp IR_str_, and is composed of three subregions (Fig. 2A). The first subregion is a novel Tn*1696*-like mercury and multidrug resistance transposon (designed Tn*6404* by Tn Number Registry). The two IRs of Tn*6404* (IR_tnp_ and IR_mer_) are interrupted by the insertion of IS*5075* into two pieces (16 bp + 22 bp), respectively. This is consistent with that IS*5075* targets a specific position in the terminal repeats of Tn21 family^24^. A similar Tn*1696*-like transposon is found in an IMP-4-encoding IncA/C2 plasmid pIMP-PH114 (KF250428) detected from a *K*. *pneumoniae* strain that was recovered from a patient who was hospitalized in the Philippines^25^ (Fig. 2B).

Tn*6404* encodes a *bla*_IMP-4_-harboring In809-like integron, replacing the In4 detected in Tn*1696*. Notably, In809 with four cassettes (*bla*_IMP-4_-*qacG2*-*aacA4*-*catB3*) is widely disseminated in *Acinetobacter* spp and Enterobacteriaceae isolates in Asia-pacific region, e.g. Hong Kong, Singapore, Australia, and Philippines^25^. Here, the In809-like integron carries a novel cassette array (*bla*_IMP-4_-*Kl.pn.I3*-*qacG2*-*aacA4*-*catB3*∆), in which a group II intron *Kl.pn.I3* is inserted into the *attC* site of the *bla*_IMP-4_ cassette (Fig. 2A). To date, various genetic context of *bla*_IMP-4_ has been identified, e.g. *bla*_IMP-4_ *-qacG-aacA4-aphA15*, *bla*_IMP-4_-*Kl.pn.I3-mobC* and *bla*_IMP-4_-*Kl.pn.I3*-*qacE*Δ-*sul1* ^7–10,16–18,22,26^. Of note, the structure *bla*_IMP-4_-*Kl.pn.I3* seems unique in isolates from China revealed by blasting in GenBank, thus could be as an epidemiological marker for *bla*_IMP-4_ detected in China.

The 5′-conserved segment (CS) of the In809-like integron is identical to that of In4 carried by Tn*1696* ^27^, encoding a complete *intI1* gene bounded by a 25-bp IRi. However, the reported *bla*_IMP-4_-*Kl.pn.I3-*harboring integrons carry a truncated *intI1* gene, mostly due to IS*26* insertions^18^. The 25-bp IRi of the In809-like integron is located between the *resII* and *resIII* sites of the *tnpR*-Tn*1696*, in the same configuration previously described in In416 (AJ704863) detected on a VIM-4- and CMY-4-encoding plasmid pCC416 in *Samonella* ^28^. The In809-like integron lacks a typical *sul1*-associated 3′-CS. Instead, a Tn*5563a*-like transposon locates at the 3′ end of the In809-like integron, and the IRR is inserted into *catB3* resulting in a truncated gene. IRL of the Tn*5563a*-like transposon is missing caused by that the *tnpR* of Tn*5563a* is replaced by a *tniR* probably via recombination.

The *mer* operon (*merRTPFADE*) of Tn*6404* is highly homologous with that of Tn5053 (L40585) (>99.9% nucleotide identity with 4-nuclotide difference), but is different with that of Tn*1696* (~84–87% nucleotide identity; a replacement of *merC* by *merF*) (Fig. 2B), suggesting that the *mer* operons of Tn*6404* and Tn*1696* have independent origin. Notably, the Tn*5563a*-like transposon together with the *mer* operon consists of a 9.4-kb module, which is almost identical to that found on pJR-1 (CP005961) from *P*. *mandelii* (2-nucleotide difference) and on pUM505 (HM560971) from *P*. *aeruginosa* (3-nucleotide difference). This indicates that the 9.4-kb module may originate from *Pseudomonas* spp. Taken together, our findings support that the ubiquity and variety of elements in the Tn*6404* is the result of frequent recombination processes.

The second subregion of MMR1 carries a module encoding a rare ESBL gene *bla*_SFO-1_, which locates adjacent to its regulator *ampR* as previously reported (Fig. 2A). The dissemination of *ampR-bla*_SFO-1_ is previously suggested to be mediated via IS*26*-composite transposon in *K*. *pneumoniae* and *E*. *cloacae* ^3,4^. However, IS*26* and/or other IS/Tn sequences were not detected here, and the genetic context of *ampR-bla*_SFO-1_ is identical to the available sequenced part (9568 bp) of an IncA/C plasmid pHD0149-2 (KJ721805) detected from an *E*. *coli* strain in China, suggesting a second dissemination mechanism harnessed by *bla*_SFO-1_.

The third subregion is a Tn*5393c* encoding *strA-strB* bracketed by two 81-bp IRs, and locates at downstream of the *bla*_SFO-1_-encoding module (Fig. 2A). The Tn*5393c* might originate from *Aeromonas salmonicida*, since it shared a high similarity with that found in pRAS2 (CP005961) from *A*. *salmonicida* (8-nucleotide difference).

MRR2 is flanked by a disrupted IR_chrA_ (22 bp) and a 19-bp IR of IS*CR1* (Fig. 2C), and is consisted of two subregions. The first subregion (IS*5075*-*chrA*-IS6100-*mphR(A)*-*mrx*-*mph(A)*-IS*26*-*aac(3′)-IId*-IS*Cfr1*-*bla*_TEM-1b_) is bracketed by a 12-bp IRR of IS*5075* and a 38-bp IR_TEM_, almost identical (3-nucleotide difference) to that detected in a Tn2-derived MRR of a *bla*_IMP-4_-harboring IncL/M plasmid pEl1573 (JX101693) from Australia^29^. It is suggested that such module can disseminate among different Inc groups via homologous recombination^25^. The second subregion of MRR2 carries an incomplete Tn*1722* transposon only encoding a truncated *tnpA* and a *tnpR*. An IS*CR1*-containing class 1 integron In37::IS*CR1* (*intI1*-*aac(6*′*)Ib*-cr-*bla*_OXA-1_-*catB3*-*arr-3*-*qacE*∆*1-sul1-*IS*CR1*) is inserted into the *res* site of Tn*1722*. Such insertion event has also been identified in an IncR plasmid pKPS30 (KF793937) and a *bla*_VIM-13_-encoding transposon (GQ396666), suggesting acquisitions of class 1 integron at a hotspot located within the *res* site of Tn*1722* ^30^. The Tn*1722*-derived MRR subregion is supposed to be obtained from pKPS30 via multiple recombination events, since they are only differed by 1 SNP and a *tnpM* deletion (Fig. 2C).

### The IS*26*-flanked composite transposon of pKP1814-3

**Figure 3:**
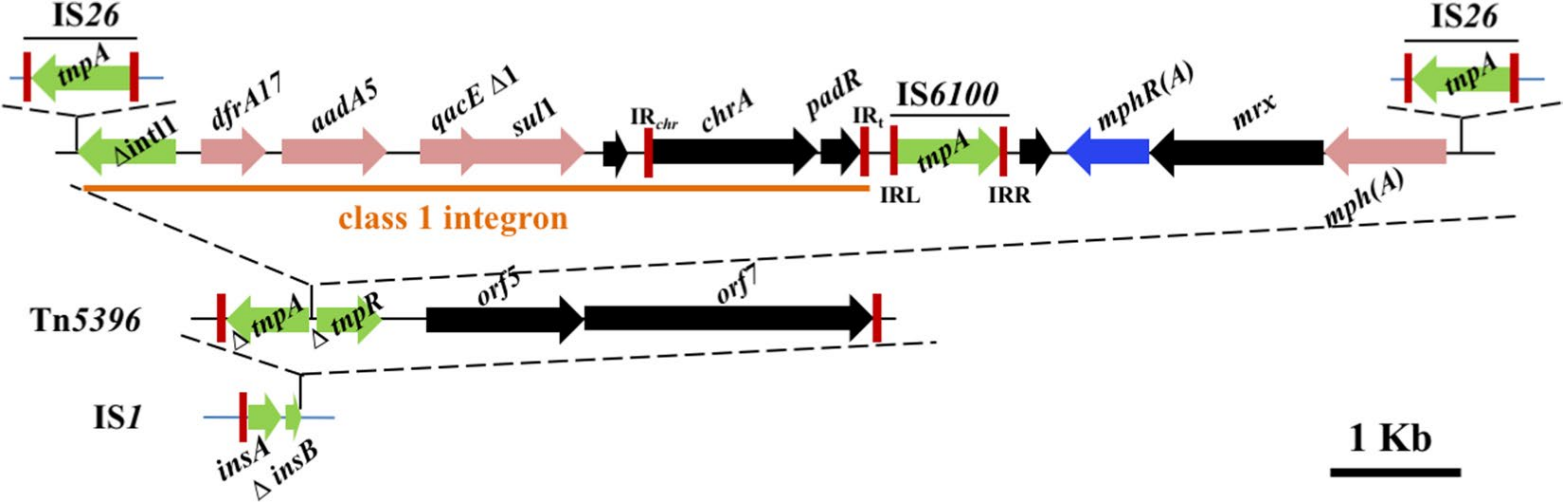
Features of the MRR of pKP1814-3. The components of the MRR are shown separately according to the obtaining order proposed. The IRR of IS*1* is missing due to the insertion of Tn*5396*. The red bars represent the IRs of IS/Tn/In. The genes are in different colours according to the classification described in Fig. 1.

The other MDR plasmid pKP1814-3 is a 95,701-bp IncFII plasmid, and was able to be co-transferred with pKP1814-1 to the azide-resistant *E*. *coli* J53. This plasmid is a hybrid of a *Shigella flexneri* plasmid pSF07201 (KJ201887) and an *E*. *coli* plasmid pCA08 (CP009233) (Fig. S1). All resistance genes of pKP1814-3 were detected in a 16-kb IS*26*-flanked composite transposon (IS*26*-∆*intI1*-*dfrA17*-*aadA5*-*qacE*∆*1-sul1-chrA-padR-*IS*6100-mphR(A)-mrx-mph(A*)-IS*26*) carrying a class 1 integron In54, of which the 5′-CS was disrupted by the insertion of IS*26* resulting in a truncated *intI1* (Fig. 3). Such IS*26*-flanked transposons have been widely disseminated in plasmids, e.g. pEC958 (HG941719), pCA08 (CP009233), and pKF3-140 (FJ876827). They are in different combinations and arrangement highlighting the role of this module in the mosaic characteristics of MRRs. It is known that IS*26*-flanked transposon could switch to be a circular form as a translocatable unit via a replicative mechanism of IS*26*, and widely disseminate by incorporating at a new location either via replicative transposition, homologous recombination or a Tnp26-catalyzed conservative reaction^31,32^. The IS*26*-flanked composite transposon was further inserted into a Tn*5396* transposon, thus is flanked upstream by a truncated *tnpA-*Tn*5396* and downstream by a truncated *tnpR*-Tn*5396*, which was nearly identical to that found in pECO-824 (CP009860) with only 1-nucleotide difference. The Tn*5396* composite transposon was further inserted into an IS*1-like* resulting in a truncated *insB* (Fig. 3), indicating multiple events involved in the acquisition of these IS/Tns here.

### The circular form of KP1814

KP1814 harbored an 18,238-bp circular form. Of note, ~58.6% of the circular form was almost identical to a region (from *chrA* to *orf*39) of pKP1814-1, and ~79% was nearly identical to the IS*26*-flanked transposon of pKP1814-3 (Fig. S2). The circular form shares a 6.78-kb module (*chrA-padR-*IS*6100-mphR(A)-mrx-mphA-*IS*26*) with pKP1814-1 and pKP1814-3. Intriguingly, a recent study detected various Tn*1548*-like circular forms in *Acinetobacter baumannii* ^33^. Taken together, this suggests an emerging role of circular forms in the dissemination of antibiotic resistance genes.

### The characterization of the virulence plasmid pKP1814-2

pKP1814-2 is a 187,349-bp circular plasmid encoding IncFIB_K_ and IncFII_K_ replicons (Fig. S3). The plasmid backbone of pKP1814-2 has a high synteny (43–46% coverage) to pKP1-19 (CP012884) and pKPN-d90 (CP015132), mainly comprising genes encoding replication/maintenance/modification (*repA*, *parA/B*, *umuC/D*, *relB/E*, *samA/B*, *ssb*, *psiA/B*, *higA*-like), F-like type IV conjugative transfer (*traMJYALEKBVCFQHGTDIX*) and other genes encoding function/hypothetical proteins (Fig. S3). The shared regions of the three plasmids were highly conserved with ~98.3% nucleotide identity (about 1500-bp difference). Numerous virulence-associated genes and one heavy-metal associated resistance gene were detected on pKP1814-2. This plasmid carried a gene encoding klebicin B, a bacteriocin involved in competitive exclusion of other bacteria to form nutritionally restricted niches^34^. Of note, an *mrk* gene cluster (*mrkABCDF*) encoding type 3 fimbria was detected. This cluster is commonly found on the chromosome of *K*. *pneumoniae*, and was occasionally identified on a few plasmids in Enterobacteriaceae. Such plasmid-borne *mrk* cluster is suggested to profoundly enhance the ability of biofilm formation and increase plasmid conjugation efficiency^35,36^. pKP1814-2 harbored a gene cluster *glgB-glgC-glgA-glgP*-*pgm* involved in the glycogen metabolic pathway, which has been linked to environmental survival, symbiotic performance and colonization, and virulence^37^. This cluster may originate from *Klebsiella* spp. as the two best matches with 100% coverage were found on pCAV1374-228 (CP011634) of a *K*. *oxytoca* strain, and on p_IncFIB_DHQP1002001 (CP016810) of a *K*. *pneumoniae* strain.

Additionally, a thermoresistance cluster, encoding heat-shock proteins ClpC, Hsp20, and ∆FtsH, was identified on pKP1814-2. This cluster could be exchanged across species as it was also detected on two *E*. *cloacae* plasmids: pENT-22e (CP009855) and pENT-4bd (CP008907). An intact *ftsH* gene was further found out of the cluster. These heat-shock proteins are known to provide protection against stressful conditions and to contribute to the bacterial pathogenesis^38–40^. The thermoresistance cluster additionally carried a *kefB* gene encoding a potassium/proton antiporter, which is known to play a role in protecting *E*. *coli* from electrophile toxicity^41^. It is assumed that those genes and gene clusters coding for carbon-source metabolism, stress response, fimbria clusters, efflux pump, and bacteriocin maybe involved in virulence properties of the host as well as in increased survival and fitness under the hospital environment, overall contributing to the successful dissemination and maintenance of these plasmids in hosts.

In conclusion, we report the complete structure of a novel plasmid carrying *bla*_SFO-1_ and *bla*_IMP-4_. Our study, along with others, raises the concern that co-existence of MDR and virulence plasmids enables to increase the fitness and viability of hosts largely challenging the clinical treatments and outcomes.

## Electronic supplementary material

Supplementary information

## References

[1] Matsumoto Y, Inoue M (1999). Characterization of SFO-1, a plasmid-mediated inducible class A beta-lactamase from *Enterobacter cloacae*. Antimicrobial Agents and Chemotherapy.

[2] Naas T, Poirel L, Nordmann P (2008). Minor extended-spectrum beta-lactamases. Clinical Microbiology and Infection.

[3] Fernández A (2011). Emergence in Spain of a multidrug-resistant *Enterobacter cloacae* clinical isolate producing SFO-1 extended-spectrum beta-lactamase. Journal of Clinical Microbiology.

[4] Guo Q (2012). Co-production of SFO-1 and DHA-1 β-lactamases and 16S rRNA methylase ArmA in clinical isolates of *Klebsiella pneumoniae*. J. Antimicrob. Chemother..

[5] Zhao J-Y (2015). Coexistence of SFO-1 and NDM-1 β-lactamase genes and fosfomycin resistance gene fosA3 in an *Escherichia coli* clinical isolate. FEMS Microbiol. Lett..

[6] Chu YW (2001). IMP-4, a novel metallo-beta-lactamase from nosocomial *Acinetobacter* spp. collected in Hong Kong between 1994 and 1998. Antimicrobial Agents and Chemotherapy.

[7] Zhao W-H, Hu Z-Q (2011). IMP-type metallo-β-lactamases in Gram-negative bacilli: distribution, phylogeny, and association with integrons. Crit. Rev. Microbiol..

[8] Espedido BA, Partridge SR, Iredell J (2008). R. bla(IMP-4) in different genetic contexts in Enterobacteriaceae isolates from Australia. Antimicrobial Agents and Chemotherapy.

[9] Roy Chowdhury P (2011). Dissemination of multiple drug resistance genes by class 1 integrons in *Klebsiella pneumoniae* isolates from four countries: a comparative study. Antimicrobial Agents and Chemotherapy.

[10] Liu Y (2009). Two clinical strains of *Klebsiella pneumoniae* carrying plasmid-borne *bla*_IMP-4_, *bla*_SHV-12_, and *armA* isolated at a Pediatric Center in Shanghai, China. Antimicrobial Agents and Chemotherapy.

[11] Mendes RE (2008). Carbapenem-resistant isolates of *Klebsiella pneumoniae* in China and detection of a conjugative plasmid (*bla*_KPC-2_ plus *qnrB4*) and a *bla*_IMP-4_ gene. Antimicrobial Agents and Chemotherapy.

[12] Li J, Hu Z, Hu Q (2012). Isolation of the first IMP-4 metallo-β-lactamase producing Klebsiella pneumoniae in Tianjin, China. Braz. J. Microbiol..

[13] Wei Z (2011). Coexistence of plasmid-mediated KPC-2 and IMP-4 carbapenemases in isolates of *Klebsiella pneumoniae* from China. J. Antimicrob. Chemother..

[14] Hawkey PM, Xiong J, Ye H, Li H, M’Zali FH (2001). Occurrence of a new metallo-beta-lactamase IMP-4 carried on a conjugative plasmid in *Citrobacter youngae* from the People’s Republic of China. FEMS Microbiol. Lett..

[15] Li B, Xu X-H, Zhao Z-C, Wang M-H, Cao Y-P (2014). High prevalence of metallo-β-lactamase among carbapenem-resistant *Klebsiella pneumoniae* in a teaching hospital in China. Can. J. Microbiol..

[16] Feng W (2016). Dissemination of IMP-4-encoding pIMP-HZ1-related plasmids among *Klebsiella pneumoniae* and *Pseudomonas aeruginosa* in a Chinese teaching hospital. Sci Rep.

[17] Lo W-U (2013). Complete sequence of an IncN plasmid, pIMP-HZ1, carrying *bla*_IMP-4_ in a *Klebsiella pneumoniae* strain associated with medical travel to China. Antimicrobial Agents and Chemotherapy.

[18] Wang Y (2017). IncN ST7 epidemic plasmid carrying bla(IMP-4) in Enterobacteriaceae isolates with epidemiological links to multiple geographical areas in China. Journal of Antimicrobial Chemotherapy.

[19] Xiao Y (2015). Bacterial-resistance among outpatients of county hospitals in China: significant geographic distinctions and minor differences between central cities. Microbes Infect..

[20] Boetzer M, Henkel CV, Jansen HJ, Butler D, Pirovano W (2011). Scaffolding pre-assembled contigs using SSPACE. Bioinformatics.

[21] Boetzer M, Pirovano W (2012). Toward almost closed genomes with GapFiller.

[22] Dolejska M (2016). High prevalence of Salmonella and IMP-4-producing Enterobacteriaceae in the silver gull on Five Islands, Australia. J. Antimicrob. Chemother..

[23] Huang T-W (2013). Complete sequences of two plasmids in a *bla*_NDM-1_-positive *Klebsiella oxytoca* isolate from Taiwan. Antimicrobial Agents and Chemotherapy.

[24] Partridge SR, Hall RM (2003). The I*S111*1 family members I*S432*1 and I*S507*5 have subterminal inverted repeats and target the terminal inverted repeats of T*n2*1 family transposons. J. Bacteriol..

[25] Ho P-L (2014). pIMP-PH114 carrying *bla*_IMP-4_ in a *Klebsiella pneumoniae* strain is closely related to other multidrug-resistant IncA/C2 plasmids. Curr. Microbiol..

[26] Yu F (2012). Outbreak of pulmonary infection caused by *Klebsiella pneumoniae* isolates harbouring *bla*_IMP-4_ and *bla*_DHA-1_ in a neonatal intensive care unit in China. J. Med. Microbiol..

[27] Essa AMM, Julian DJ, Kidd SP, Brown NL, Hobman JL (2003). Mercury resistance determinants related to T*n2*1, T*n169*6, and T*n505*3 in enterobacteria from the preantibiotic era. Antimicrobial Agents and Chemotherapy.

[28] Colinon C, Miriagou V, Carattoli A, Luzzaro F, Rossolini GM (2007). Characterization of the IncA/C plasmid pCC416 encoding VIM-4 and CMY-4 beta-lactamases. Journal of Antimicrobial Chemotherapy.

[29] Partridge SR, Ginn AN, Paulsen IT, Iredell JR (2012). pEl1573 Carrying *bla*_IMP-4_, from Sydney, Australia, is closely related to other IncL/M plasmids. Antimicrobial Agents and Chemotherapy.

[30] Compain F (2014). Complete nucleotide sequence of two multidrug-resistant IncR plasmids from *Klebsiella pneumoniae*. Antimicrobial Agents and Chemotherapy.

[31] Harmer CJ, Moran RA, Hall RM (2014). Movement of IS*26*-associated antibiotic resistance genes occurs via a translocatable unit that includes a single IS*26* and preferentially inserts adjacent to another IS*26*. MBio.

[32] Harmer, C. J. & Hall, R. M. IS*26*-Mediated Formation of Transposons Carrying Antibiotic ResistanceGenes. *mSphere* 1 (2016). 10.1128/mSphere.00038-16 PMC4894685 27303727

[33] Karah N (2016). Novel Aminoglycoside Resistance Transposons and Transposon-Derived Circular Forms Detected in Carbapenem-Resistant *Acinetobacter baumannii* Clinical Isolates. Antimicrobial Agents and Chemotherapy.

[34] Riley MA, Pinou T, Wertz JE, Tan Y, Valletta CM (2001). Molecular characterization of the klebicin B plasmid of *Klebsiella pneumoniae*. Plasmid.

[35] Ong C-LY (2008). Identification of type 3 fimbriae in uropathogenic *Escherichia coli* reveals a role in biofilm formation. J. Bacteriol..

[36] Norman A, Hansen LH, She Q, Sørensen SJ (2008). Nucleotide sequence of pOLA52: a conjugative IncX1 plasmid from *Escherichia coli* which enables biofilm formation and multidrug efflux. Plasmid.

[37] Eydallin G (2010). Genome-wide screening of genes whose enhanced expression affects glycogen accumulation in *Escherichia coli*. DNA Res..

[38] Ventura M (2006). How high G + C Gram-positive bacteria and in particular bifidobacteria cope with heat stress: protein players and regulators. FEMS Microbiol. Rev..

[39] Neckers L, Tatu U (2008). Molecular chaperones in pathogen virulence: emerging new targets for therapy. Cell Host Microbe.

[40] Langklotz S, Baumann U, Narberhaus F (2012). Structure and function of the bacterial AAA protease FtsH. Biochim. Biophys. Acta.

[41] Khare G, Reddy PV, Sidhwani P, Tyagi AK (2013). KefB inhibits phagosomal acidification but its role is unrelated to *M. tuberculosis* survival in host. Sci Rep.

