# Supplementary material for: A novel Tn1696-like composite transposon (Tn6404) harboring blaIMP-4 in a Klebsiella pneumoniae isolate carrying a rare ESBL gene blaSFO-1
Source: Sci Rep. 2017 Dec 11;7:17321. doi: 10.1038/s41598-017-17641-2 (PMC5725488; doi:10.1038/s41598-017-17641-2)
Supplement: Supplementary file 1 — Supplementary information [file 41598_2017_17641_MOESM1_ESM.doc]

**A novel Tn*1696*-like composite transposon (Tn*6406*) harboring *bla*_IMP-4_ in a *Klebsiella pneumoniae* isolate carrying a rare ESBL gene *bla*_SFO-1_**

Kai Zhou^1^, Wei Yu^1,3^, Ping Shen^1^ Haifeng Lu^1^, Baohong Wang^1^, John W. A. Rossen^2^, Yonghong Xiao^1*^

^1^ State Key Laboratory for Diagnosis and Treatment of Infectious Diseases; Collaborative

Innovation Center for Diagnosis and Treatment of Infectious Diseases, the First Affiliated

Hospital of Medicine School, Zhejiang University, Hangzhou, China;

^2^ Department of Medical Microbiology, University of Groningen, University Medical Center Groningen, Groningen, Netherlands;

^3^Department of Infectious Diseases, Zhejiang Provincial People’s Hospital, Hangzhou, China.

**Table S1 MICs of antibiotic agents for KP1814 and transconjugants**

|  |  | MIC (mg/L) | |
| --- | --- | --- | --- |
|  | KP1814 | *E.coli* J53 Azi^R^ (pKP1814-1+ pKP1814-3) | *E.coli* J53 Azi^R^ |
| Ampicillin | ≥32 (R) | ≥32 (R) | 2 (S) |
| Ampicillin-sulbactam | ≥32 (R) | ≥32 (R) | 1 (S) |
| Piperacillin-tazobactam | 64 (R) | 8 (R) | 1 (S) |
| Cefazolin | ≥64 (R) | ≥64 (R) | 1 (S) |
| Ceftriaxone | ≥64 (R) | 32 (R) | 0.064 (S) |
| Cefotetan | ≥64 (R) | ≥64 (R) | 0.125 (S) |
| Ceftazidime | ≥64 (R) | ≥64 (R) | 0.064 (S) |
| Cefepime | 16 (R) | 8 (R) | 0.064 (S) |
| Aztreonam | ≥64 (R) | 4 (S) | 0.125 (S) |
| Ertapenem | ≥8 (R) | 4 (R) | 0.064 (S) |
| Imipenem | 2 (S) | 4 (S) | 0.125 (S) |
| Amikacin | ≤2 (S) | ≤2 (S) | 0.25 (S) |
| Gentamicin | ≥16 (R) | ≥16 (R) | 0.5 (S) |
| Tobramycin | 8 (R) | 4 (S) | 0.5 (S) |
| Ciprofloxacin | ≤0.25 (S) | ≤0.25 (S) | 0.032 (S) |
| Levofloxacin | ≤0.25 (S) | ≤0.25 (S) | 0.032 (S) |
| Nitrofuranton | 64 (S) | ≤16 (S) | 2 (S) |
| Trimethoprim/sulfamethoxazole | ≥320 (R) | ≥320 (R) | 0.5 (S) |

**Figure S1. The genetic map of pKP1814-3.** A circular representation of pKP1814-3 was generated by using DNAplotter. From the inside outward, the first four circles represent GC skew, GC content, the regions with over 50% nucleotide identity to pSF07201 (KJ201887), and pCA08 (CP009233), respectively. The last two circles represent the genetic features in counterclockwise and clockwise directions, respectively. Genes are classified by different colours as shown in the legend, and the names of some genes are shown. The best blastn hit of IS*26*-flanking composite transposon to pECO-824 (CP009860) is shown as the brown arch.

**Figure S2. Syntany analysis of pKP1814-1, pKP1814-3, and the circular form.** The genes are classified by different colours as shown in Figure 1.

**Figure S3. The genetic map of pKP1814-2.** A circular representation of pKP1814-2 was generated by using DNAplotter. From the inside outward, the first four circles represent GC skew, GC content, the regions with over 50% nucleotide identity to pKP1-19 (CP012884), and pKPN-d90 (CP015132), respectively. The last two circles represent the genetic features in counterclockwise and clockwise directions, respectively. Genes are classified by different colours as shown in the legend. The names of some genes are shown. The best blastn hit of virulence-associated regions are shown as the brown arches.
